# The Impact of 850,000 Years of Climate Changes on the Structure and Dynamics of Mammal Food Webs

**DOI:** 10.1371/journal.pone.0106651

**Published:** 2014-09-10

**Authors:** Hedvig K. Nenzén, Daniel Montoya, Sara Varela

**Affiliations:** 1 Département des sciences biologiques, Université du Québec à Montréal, Montréal, Québec, Canada; 2 School of Biological Sciences, Life Sciences Building, University of Bristol, Bristol, United Kingdom; 3 Department of Ecology, Faculty of Science, Charles University, Prague, Czech Republic; University of York, United Kingdom

## Abstract

Most evidence of climate change impacts on food webs comes from modern studies and little is known about how ancient food webs have responded to climate changes in the past. Here, we integrate fossil evidence from 71 fossil sites, body-size relationships and actualism to reconstruct food webs for six large mammal communities that inhabited the Iberian Peninsula at different times during the Quaternary. We quantify the long-term dynamics of these food webs and study how their structure changed across the Quaternary, a period for which fossil data and climate changes are well known. Extinction, immigration and turnover rates were correlated with climate changes in the last 850 kyr. Yet, we find differences in the dynamics and structural properties of Pleistocene *versus* Holocene mammal communities that are not associated with glacial-interglacial cycles. Although all Quaternary mammal food webs were highly nested and robust to secondary extinctions, general food web properties changed in the Holocene. These results highlight the ability of communities to re-organize with the arrival of phylogenetically similar species without major structural changes, and the impact of climate change and super-generalist species (humans) on Iberian Holocene mammal communities.

## Introduction

Climate change is one of the major drivers affecting the diversity, composition, structure and functioning of ecological communities. Species respond in different ways to climate change which directly affects their persistence within the food web and, consequently, the composition and structure of the community [1], [2]. Although evidence of the impacts of climate change mostly comes from studies of modern communities, life on Earth has experienced several climatic perturbations in the past and those changes impacted the composition and structure of ancient communities in similar ways. Understanding how ancient food webs responded to past climate provides information about how communities reorganize across time, and how food webs could respond to contemporary climate change [3], [4].

Because the Quaternary fossil record is extensive and the climate changes during this period are relatively well understood, the examination of the Quaternary fossil record could be key to understanding long-term dynamics and structure of ancient biological communities [4]. Climate in the Quaternary is characterized by cyclic climatic changes, oscillating from cold, dry glacial scenarios to warm, wet interglacial scenarios [5]. Changes in annual mean temperature inferred from oxygen isotopes range from an increase of 5°C during the warm scenarios to a decrease of −11°C in the extreme glacial periods [6]. This sequence of successive glacial and interglacial periods had direct and indirect effects on natural communities by forcing species to migrate [4], eliminating species [7], introducing new species, and shaping several broad diversity patterns that we observe today (e.g. [8]).

In the Iberian Peninsula, fossil records show that the composition of species within communities changed during the Quaternary following climate changes (e.g. [9]). Whereas some species went extinct after inhabiting the Iberian Peninsula for long time periods (e.g. *Bos primigenius*, *Crocuta crocuta*), others remained for more than one million years (*Cervus elaphus*, *Sus scrofa*), and certain species appeared for short periods (*Cuon alpinus*, in the Middle Pleistocene; or *Coelodonta antiquitatis*, in the Last Glacial Maximum). Quaternary climate changes co-occurred with extinctions, migrations and the arrival of new species in the Iberian Peninsula that have likely impacted the structure of mammal communities living in this region.

In spite of the observed long-term species changes during the Quaternary, few studies have analysed long series of species turnovers (but see [10]), even fewer have investigated the structure of the mammal communities through time [7], [11], [12] and none have analyzed food web dynamics across the Quaternary. Analyzing food webs can help us to identify changes in biotic interactions, beyond counting the number of species present in the community [13], [14]; these changes can alter the functioning of food webs and ecosystems [15], and affect their future stability and persistence [16], [17]. However, food web approaches in paleoecological studies are scarce, and therefore we know little about how past climate changes have affected the long-term dynamics and food web structure of biological communities at regional scales, and to what extent these communities re-organize in response to these impacts (but see [18]–[21] for temporal food web studies). The reason for this paucity of studies is twofold. First, extending the approaches used in food web theory to ancient communities has been limited by the incompleteness or lack of fossil data. In particular there is little fossil evidence that can be used to establish trophic links, compared to highly-resolved modern networks where changes in feeding interactions can be directly observed (e.g. [22]).

Second, the information from one single fossil site is limited for studies at the community scale where several species are considered. Because of a number of biases that affect the fossil record [23], individual fossil sites often lack species from the regional species pool (the diversity of species at a spatial scale larger than the individual fossil site) and are not an appropriate source of data to construct ancient regional food webs. Here we overcome these limitations by using large-scale cumulative food webs, that are constructed by using fossil information from different sites, and are thus appropriate for comparing food webs over time or space [20]. Moreover, large-scale cumulative webs do not depend on single fossil site records, and minimize problems of undersampling or collection bias [23], [24].

In this work, we assemble six large mammal communities in the Iberian Peninsula spanning 850,000 years (Pleistocene and Holocene time periods). To obtain a complete species pool for large Iberian mammals, communities were constructed using published data from 71 fossil sites. Thus, the results presented here describe how large mammal communities at a regional scale changed during the Quaternary climate changes. The aim of this study is to explore the long-term and community structure of a large mammal food web across 850,000 years of climate changes. We specifically address the following questions: (a) how have Quaternary mammal communities changed in terms of dynamics (species extinctions, immigrations, and turnover rates) and food web structure?, and (b) are the changes in the structure and dynamics of these communities associated with Quaternary climate changes?

## Methods

### Fossil data and food web construction

We assembled six different large mammal food webs across the Quaternary: one for the Early Pleistocene (EP; around 850,000 years before present), one for the Middle Pleistocene (MP; around 450 kyr BP), plus two food webs during the Late Pleistocene, one for the last interglacial maximum, LIM; 120 kyr BP, and one for the last glacial maximum, LGM; 21 kyr BP), and two food webs for the Holocene (H; 10 kyr BP and present, P). For each time, we identified the large mammals (>20 kg), including hominids, that were present using fossil records from several Iberian fossil sites (details about sites and references in Table S1 and figure S1 in File S1). The taxonomy has been revised and unified following [25]. The resulting communities are not observed local communities, but regional food webs constructed using data from 71 fossil sites within the Iberian Peninsula. Large mammals are highly mobile and we assume that their geographic ranges covered the entire Iberian Peninsula. We use the number of glacial cycles between each community, MIS/OIS boundaries (Marine Isotope Stages, or Oxygen Isotope Stages [26]) as a proxy for climatic changes. We assume that a higher number of cycles represent a higher number of recovery and reorganisation periods for the mammal communities, indicating that they experienced greater stress.

**Figure 1:**
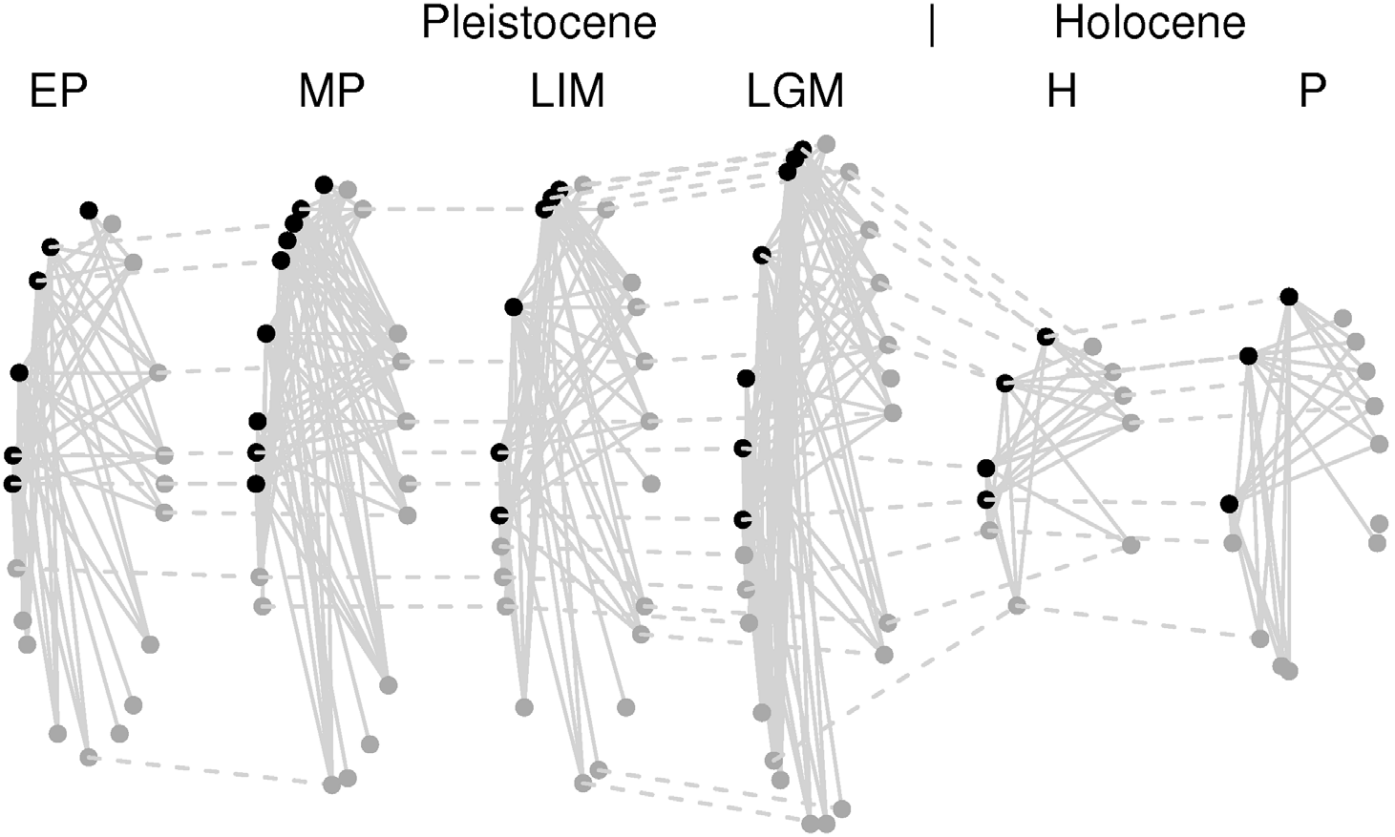
Large mammal food webs of the Iberian Peninsula during the Quaternary. Early Pleistocene (EP; 850,000 years before present, or 850 ky BP), Middle Pleistocene (MP; 450 kyr BP), Last Interglacial Maximum (LIM; 120 kyr BP), Last Glacial Maximum (LGM; 21 kyr BP), Early Holocene (H; 10 kyr BP) and present (P). Each species (black for predator, dark grey for prey) is a node, and each link indicates a trophic interaction. Horizontal links indicate that the species persists between communities.

Next we determine the trophic links between the species present in each community (figure 1, figure S2 in File S1). This is the most challenging step since diet evidence of extinct species is rare and interactions cannot be observed in the field. Species eaten by Pleistocene carnivores are classically identified from indirect clues, such as cut marks, teeth marks or stone tools marks in the fossil bones [27]. There are methods to distinguish carnivorous vs. herbivorous species [28], [29], and to identify ancient species diet requirements using stable isotopes [21] and DNA from coprolites [30]. Here, food web links were designated following three criteria: (i) spatio-temporal co-occurrence as shown by fossil record data (Tables S1, S2 in File S1), (ii) body-size relationships (Table S3 in File S1), and (iii) actualism (applying current species diet to infer past trophic links). The key role of body-size in determining feeding links and structuring food webs is broadly accepted [31]. We complement link information by using actualism, which can be applied to all Quaternary carnivores (e.g. human diet cannot be predicted by body weight alone). Actualism diets follow [32]. The networks analysed here are bipartite and consist of two trophic levels (predators and herbivores/prey).

### Community and food web analysis

We explore community long-term dynamics by calculating the number of extinctions, immigrations and species turnover between communities. Extinction and immigration were estimated as the number of species that were lost or gained in each transition. We use Sørensen's dissimilarity index to estimate species turnover rates between the communities [33].

We also explore the possibility that extinction and immigration are non-random by using two plausible hypotheses based on phylogeny and body-size. For immigrating species, we test whether newly immigrated species are phylogenetically similar to the species that went extinct in the preceding time step. Following the phylogenetic conservatism hypothesis, new immigrants are more likely to replace extinct species if they are closely related within the evolutionary tree (because of functional similarity), a phenomenon that seems to be widespread across evolutionary time [34]. To test if species were replaced by closely related species, we count the number of times a species that went extinct was replaced by a species within the same genus (Table S2 in File S1). In the absence of a complete phylogeny this gives a simple measure of phylogenetic relatedness and functional similarity, as closely related species often fulfil the same function in an ecosystem [34]–[36]. Second, as large animals are likely to go extinct first [37], [38], we test whether the body-size distribution of extinct species was random across time. For each food web we randomly remove the same number of species that were observed to go extinct in the next time step. After repeating this 10,000 times, we compare the mean body weight of the randomized and observed food webs.

To examine food web structure we use standard metrics: species richness, number of links and connectance (links/species^2^) [39]. Two other food web properties were analysed: vulnerability (mean number of predators per prey), and generality (mean number of prey per predator) [40]. To further characterize generality we sort the number of links per prey and predator and plot the relationship for each food web. Because network structure is related to its stability, we analysed community stability by examining (i) the robustness of ancient food webs to species loss [41]–[43], and (ii) nestedness [44]. The robustness index measures the topological or structural stability of the food web by simulating how random removal of prey (or predators) induces secondary extinction among the predators (or prey) [43], [45]. Robustness is measured as the area under the curve of the number of species being removed against the number of secondary extinctions, and ranges from 0 to 1, with high values representing more robust communities (the number of secondary extinctions is lower). We calculate community robustness for removal of both prey and predators individually. Nestedness measures the degree to which the diets of consumers are proper subsets of other, more generalist consumers. The nestedness algorithm used here is based on the nestedness temperature of the interaction matrix, and ranges from 0 which indicates high nestedness, to 100 which indicates no nestedness [46], [47].

Finally, we examine the relationships between climate change, species turnover and food web properties. Controlled, replicated experiments cannot be conducted in paleoecological studies, but we can explore whether food web changes in ancient communities are associated to climatic changes in the past. We test if the number of glacial cycles between each community and the observed changes in species composition are related to changes in food web metrics, using the 15 unique pairwise comparisons between all six food webs. Given that non-linear responses of natural communities to climate changes are common (e.g. [48]), we assume that the relationship between the number of glacial cycles, species turnover and food web properties are non-linear, and for that reason we use the Spearman correlation index.

## Results

### Changes in Quaternary mammal food webs over the last 850,000 years

**Figure 2:**
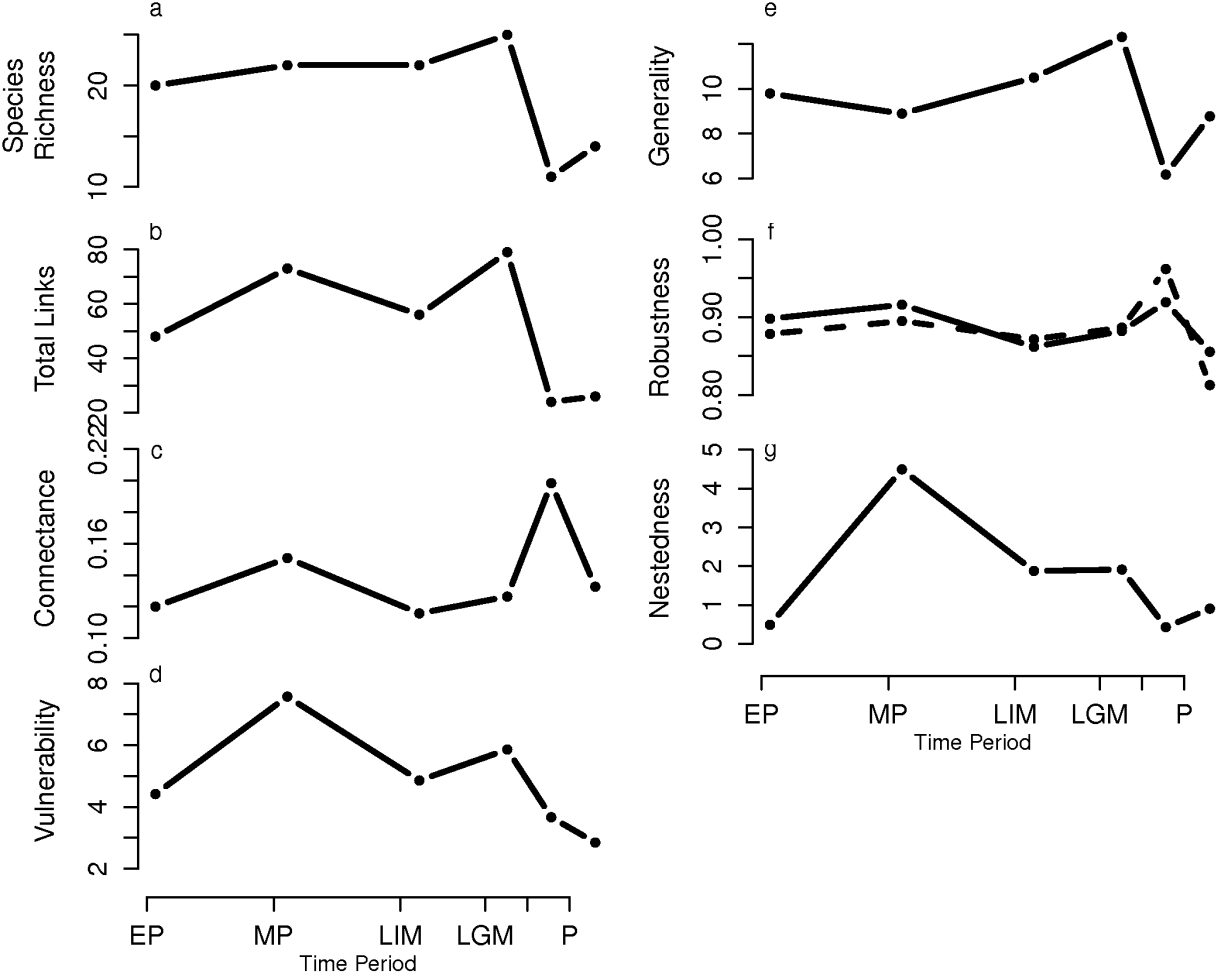
Food web properties at each time step. (a) Species richness (number of species), (b) total links (total number of links or trophic interactions between species), (c) connectance (links/species^2^), (d) vulnerability, (e) generality, (f) robustness against removal of prey (solid line) and predators (dotted line), (g) nestedness. The dates are the same as in figure 1. The x-axis is proportional to the time between each community.

Pleistocene communities comprise 20–25 species >20 kg (figure 2a), a figure that falls within the range of values reported in comparative analyses of modern [49] and ancient food webs [20], [21]. The complexity of these ancient webs is also similar to that observed in modern mammal food webs in Africa where megafauna did not go extinct in the Holocene [50].

**Figure 3:**
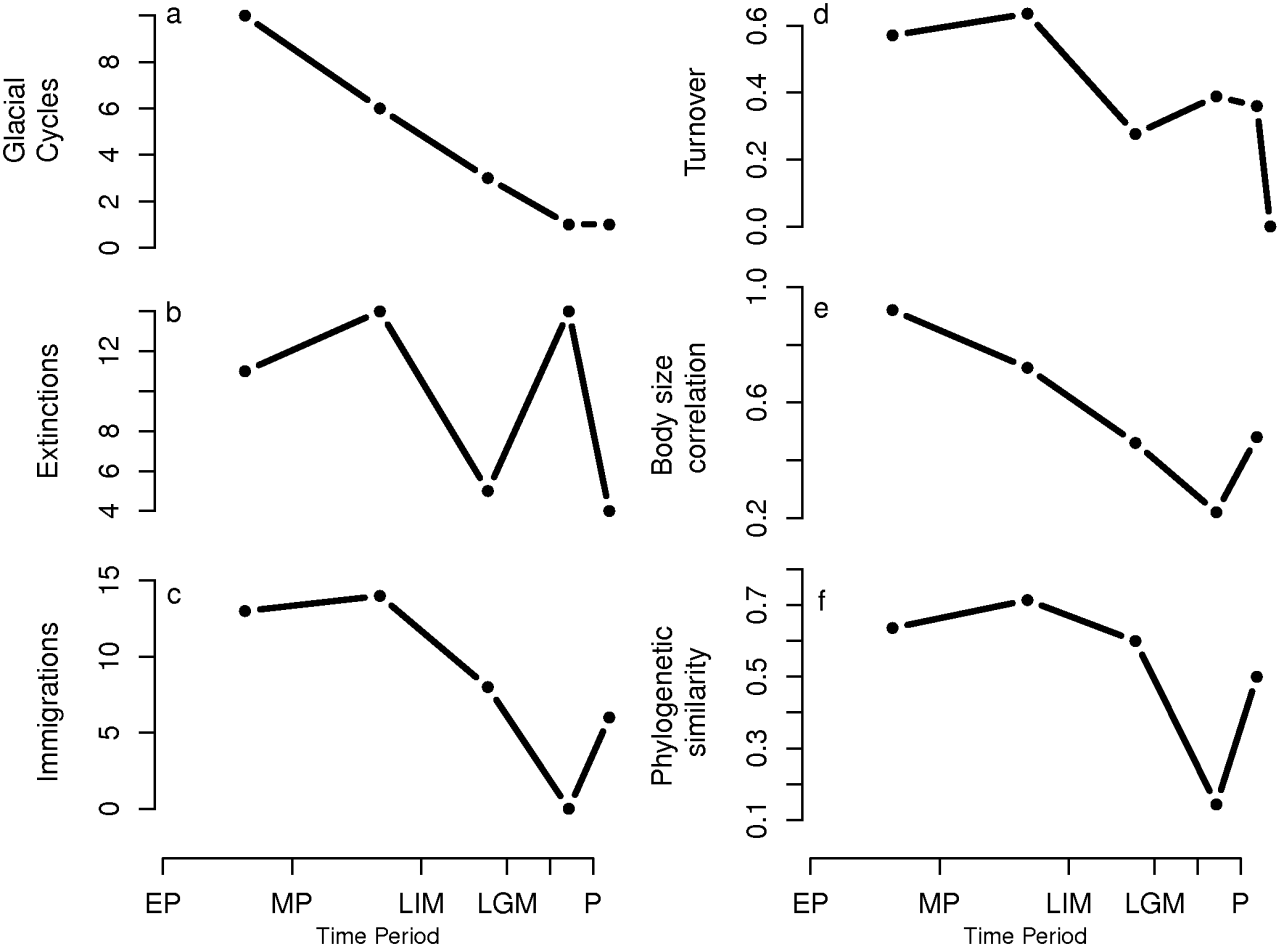
Food web structural changes at each time step. (a) Number of glacial cycles, (b) extinct species and (e) immigrating species, (f) species turnover, (g) correlation between observed and randomly expected body size distribution of extinct species and (f) phylogenetic similarity. The dates are the same as in figure 1.

During the Pleistocene the rates of extinction were high, (figure 3b, except the LIM-LGM transition), with one species disappearing every 20–36 kyr. Extinction analysis showed no correlation between species’ body-size and extinction probability (figure 3e). The number of new species entering Pleistocene communities equalled or exceeded the number of species that went extinct (figure 3c), with one new species entering the Pleistocene food webs every 12–36 kyr. This suggests that extinct species were replaced by new species at similar rates, and that the overall number of species in Pleistocene food webs did not change. Consistent with the phylogenetic conservatism hypothesis, a high proportion of these new species were phylogenetically similar to the extinct species (figure 3f). Despite the high turnover rates (figure 3d), the general diversity and food web properties of the Pleistocene communities remained relatively constant across almost 500 kyr.

The dynamic stability observed in Pleistocene mammal communities changed in the Holocene. Extinction rates dramatically increased (one extinct species every 830 years, figure 3b). These extinctions are non-random (figure 3e), and especially affect the least connected specialist predators (figure S3 in File S1) and species with large body-sizes (figure S4 in File S1). Immigration rate dropped (figure 3c), and, consequently, extinct species were not replaced by new ones. Intriguingly, no predator mammals entered into the food web during the Holocene and the few immigrant species were phylogenetically unrelated (figure 3f). These processes caused a reduction in the number of species at all trophic levels that affected food web structure in two ways: (i) connectance increased, and (ii) generality and vulnerability decreased. The observed differences in other food web properties between Holocene and Pleistocene communities may be a direct consequence of increases in connectance following a reduction in network size and the loss of specialist predators.

Regardless of the changes in community size between Pleistocene and Holocene communities, the robustness of large mammalian communities to secondary extinctions of both prey and predators remained constant across the Quaternary period (figure 2f). We found no correlation between connectance and robustness, nor between species richness and robustness across the Quaternary period (Spearman correlations, p>0.05). Nestedness patterns, on the other hand, have fluctuated but fall within high nestedness values (figure 2g).

### The role of climate change in the dynamics and structure of Quaternary mammal food webs

Overall, turnover rates of Iberian mammal species across the Quaternary are associated with the pattern of glacial cycles (table 1). Quaternary climate changes are positively correlated with species turnover, extinction and immigration rates across all mammal communities. However, glacial-interglacial events show no significant relationship with food web properties, which suggests that climate changes across the Quaternary were not associated with changes in general structure of mammalian communities. We also assessed whether climate change is indirectly associated with food web properties through changes in species composition, yet no significant relationship was observed between species turnover rates and food web metrics (table 2). This suggests that the structure of the Quaternary mammal food webs is independent of observed species turnover rates, and that most of the changes in the food web properties between Pleistocene and Holocene mammal communities are associated with the loss of specialist predators and increases in connectance following a non-random reduction in network size between these two periods of time.

**Table 1:** Spearman’s rank correlation coefficient between climate change (number of glacial-interglacial cycles between scenarios) and species and food web structure parameters for all communities. ρ = Spearman correlation.

Climate change vs.		ρ	S	p
Species	*Species turnover*	0.92	47.54	<0.001
	*Extinction*	0.74	140.24	0.001
	*Immigration*	0.65	193.36	0.008
Food web structure	Species Richness	−0.15	654.77	0.585
	Number of food web links	−0.15	646.00	0.584
	Connectance	−0.24	692.00	0.397
	Predator/prey ratio	−0.07	598.00	0.812

**Table 2:** Spearman’s rank correlation coefficient between species and food web structure parameters for all communities.

Species turnover vs.		rho	S	p
Food web structure	Species Richness	−0.06	594.33	0.828
	Number of links	−0.07	600.03	0.800
	Connectance	−0.34	752.17	0.210
	Predator/prey ratio	−0.11	623.05	0.689

## Discussion

Iberian large mammal communities have experienced important dynamical changes across the Quaternary. Extinction, immigration and turnover rates were highly correlated with climate changes in the last 850 kyr, yet food web properties were not significantly altered following these climate changes. Our results suggest that glacial-interglacial cycles are associated with changes in community dynamics, and highlights the ability of communities to re-organize with the arrival of phylogenetically similar species without major changes in food web properties. As a result, ancient mammal food webs were dynamically stable and able to recover from climatic perturbations even though many species went extinct.

### Changes in large mammal food web structure and dynamics

Our results show differences in the dynamics and structure of Pleistocene *versus* Holocene mammal communities on the Iberian Peninsula. In the Pleistocene, extinctions were common and affected species irrespective of their body-size. Extinct species in the food web were replaced by newly arriving species that were closely related in the phylogenetic tree, so extinction and immigration rates were balanced. Consequently, food web properties remained unaltered and Pleistocene communities remained structurally stable despite the species identities. These results support the phylogenetic conservatism hypothesis [34], by which new immigrants are more likely to replace extinct species if they are closely-related within the evolutionary tree, as closely related species often fulfil the same function in the ecosystem. However, the extinction-immigration patterns of the Pleistocene are not reproduced in Holocene communities, which contracted as a result of higher extinction rates (56% of species went extinct from the early Pleistocene to Holocene) and low replacement rates. This reduction in community size and loss of specialist species increased connectance, which in turn affected other properties of the food web.

After more than 800,000 years of relative stability, food web properties changed in two steps in the Holocene: species richness and generality first decreased and then increased. The first step was caused by the extinction of large mammals after the Last Glacial Maximum (21–10 kyr BP), when prey such as *Mammuthus primigenius* (woolly mammoth) or *Megaloceros giganteus* (Irish elk) went extinct, followed by large carnivores like *Panthera spelaea* (lion). This is consistent with the observation that modern mammalian communities are remnants of larger Pleistocene mammal communities [51]. This reduction in community size is observed in contemporary mammal communities, and affected food web properties by reducing generality (fewer prey species) and increasing connectance. Theoretical studies suggest that spatially coupled food webs are especially sensitive to the loss of large animals [52], and there is evidence showing that the loss of megaherbivores such as mammoths had large impacts on ecosystem function [53], [54]. Consequently, the loss of large mammals may have also affected the ecosystem functions provided by Holocene communities.

In the second step, during the Holocene only human-introduced herbivores, such as *Bos taurus* (cattle) or *Equus caballus* (horse) appeared as new species in the food webs. These introductions slightly increased the number of species and the mean number of prey species per predator, the latter being also a consequence of the existence of generalist predator species, especially the humans. In contrast to Pleistocene communities, the introduced species were not phylogenetically related to the extinct species, and this has likely impacted ecosystem functioning, as certain ecosystem functions may not have been completely replaced by phylogenetically related species.

### Robustness and nestedness

Our analysis of robustness reveals across-time similarities in Pleistocene and Holocene communities. Despite the significant extinction and turnover rates across the Quaternary, and the reported changes in community size and food web connectivity, the robustness of mammal communities remained fairly constant for nearly one million years. On the other hand, all our mammal food webs present highly nested structures. Simulations have shown recently that nestedness is a destabilizing force in predator-prey food webs [55], and its absence in other ancient food webs may have promoted stability during the successive climatic changes that occurred in the past [21]. Although the combination of high robustness and high nestedness may have resulted in a neutral effect on stability, nested structures may have also acted as a buffer to secondary extinctions [56], so the interpretation of historical patterns of nestedness of Quaternary mammal communities is not straightforward.

The high nestedness reported in Quaternary mammal communities is explained by the presence of highly generalist predators. Both theoretical and empirical evidence show that super-generalist species become central nodes (most connected species) in the core of the nested community and may increase the overall nestedness [57]. Perturbation events usually favour generalist over specialist species, a pattern observed both in paleoecological [58] and contemporary communities [59], and this contributes to increase the generalist:specialist ratios. Quaternary mammal food webs were no exception to this rule and, as a result of the long-term dynamics following glacial-interglacial cycles, comprised highly generalist species. Specifically, the Holocene period experienced a population growth of the super-generalist anatomically modern humans. Humans not only have a broad diet (figure S2 in File S1), but also introduced domesticated mammals into the food web, intensified the generality and contributed to the high connectivity of Iberian mammal food webs. Anatomically modern humans have been present in Spain since 42 kyr BP, but with a small population size [60]. Other hominids such as *Homo neanderthalensis* were present earlier, but our results suggest that they had a smaller impact on food webs compared to Holocene populations of *Homo sapiens*.

The high impact of omnivore humans on food webs has also been found in contemporary communities. A recent study explored a spatial (rather than temporal) gradient of human impacts in Serengeti food webs, average body mass and species richness also decreased with increasing human impact. Food webs tended to be more generalist following the human-induced extinction of the least-connected, most specialized species [61]. In contrast to Quaternary mammal communities, the Serengeti food web harbours more trophic levels and large predators, which results in a higher species number and lower connectance compared to our food webs.

## Conclusion

Paleoecological community studies provide insight into the relationships between biological communities and perturbations such as climate change over long time scales. This work presents a first attempt to track temporal changes in food webs over long time periods. Although the spatial and temporal scales are coarse, the patterns revealed are significant. We found that large mammal communities in the Pleistocene were able to re-organize despite the high extinction rates reported in the last 850,000 years. However, our results indicate that Holocene mammal communities experienced changes that were related to the arrival of new species introduced in the communities by humans (cattle and game species). Future work should aim at investigating the actual changes in temperature at local scales and the duration of those cycles; this information could be used to make cross-comparisons of the species’ extinction rates within and among continents, and their effects on ancient food webs, and to understand the mechanisms driving the likely spatial differences in the extinction rates.

Collectively, our results suggest differences between the Pleistocene and Holocene time periods in the structure and dynamics of large mammal food webs. These conclusions have consequences for understanding the current-day sixth extinction event, and to what extent modern communities will be able to re-organize during current climate change. Not only are current rates of change in climate high, but also they interact with other drivers of extinction such as habitat loss and overexploitation, and the feedbacks generated by these processes and biotic interactions may result in higher extinction rates than those expected only from climate change [62], [63].

## Supporting Information

File S1 **Table S1,** Reference sources used for determining the presence of species in each of the 71 sites. **Table S2,** The species present (indicated by 1) at each period. Weight categories: 1 = <45 kg, 2 = 45–90 kg, 3 = 90–360 kg, 4 = 360–1000 kg, 5 = >1000 kg. Phylogenetic replacement categories: 0 = Not replaced by a species in the same genus in the next time period, 1 = Replaced by a species in the next time period, 2 = Species still present. **Table S3,** Prey weight classes. This information, together with spatio-temporal co-occurrence and actualism (see main text) was used to establish the links between species in the food web. **Figure S1,** Geographic location on the Iberian Peninsula of the Quaternary fossil sites used for constructing the ancient food webs. **Figure S2,** Large mammal food webs of the Iberian Peninsula during the Quaternary. (a) Early Pleistocene (850,000 years before present, or 850 ky BP); (b) Middle Pleistocene (450 ky BP); (c) Last Interglacial Maximum (120 ky BP); (d) Last Glacial Maximum (21 ky BP); (e) Early Holocene (10 ky BP); and (f) Present. Each node (green for prey, red for predator) is a species, and each link indicates a trophic interaction. **Figure S3,** Number of prey per predator in each time period, with species sorted in descending order. The time periods are the same as in figure 1. **Figure S4,** A random extinction experiment demonstrate that the distribution of the number of links, connectance and link density observed Holocene food web is not expected by chance. 1000 food webs have been created extracting randomly 11 species from last glacial maximum food web (the observed number of extinct species in the Holocene).
(DOCX)
