# Supplementary material for: The Impact of 850,000 Years of Climate Changes on the Structure and Dynamics of Mammal Food Webs
Source: PLoS One. 2014 Sep 10;9(9):e106651. doi: 10.1371/journal.pone.0106651 (PMC4160162; doi:10.1371/journal.pone.0106651)
Supplement: File S1 — Table S1, Reference sources used for determining the presence of species in each of the 71 sites. Table S2, The species present (indicated by 1) at each period. Weight categories: 1 = <45 kg, 2 = 45–90 kg, 3 = 90–360 kg, 4 = 360–1000 kg, 5 = >1000 kg. Phylogenetic replacement categories: 0 = Not replaced by a species in the same genus in the next time period, 1 = Replaced by a species in the next time period, 2 = Species still present. Table S3, Prey weight classes. This information, together with spatio-temporal co-occurrence and actualism (see main text) was used to establish the links between species in the food web. Figure S1, Geographic location on the Iberian Peninsula of the Quaternary fossil sites used for constructing the ancient food webs. Figure S2, Large mammal food webs of the Iberian Peninsula during the Quaternary. (a) Early Pleistocene (850,000 years before present, or 850 ky BP); (b) Middle Pleistocene (450 ky BP); (c) Last Interglacial Maximum (120 ky BP); (d) Last Glacial Maximum (21 ky BP); (e) Early Holocene (10 ky BP); and (f) Present. Each node (green for prey, red for predator) is a species, and each link indicates a trophic interaction. Figure S3, Number of prey per predator in each time period, with species sorted in descending order. The time periods are the same as in figure 1. Figure S4, A random extinction experiment demonstrate that the distribution of the number of links, connectance and link density observed Holocene food web is not expected by chance. 1000 food webs have been created extracting randomly 11 species from last glacial maximum food web (the observed number of extinct species in the Holocene). (DOCX) [file pone.0106651.s001.docx]

**Electronic Supplementary Material for:**

**THE IMPACT OF 850,000 YEARS OF CLIMATE CHANGES ON THE STRUCTURE**

**AND DYNAMICS OF MAMMAL FOOD WEBS**

Hedvig K Nenzén^1^, Daniel Montoya^2^ and Sara Varela^3^

^1^Département des sciences biologiques, Université du Québec à Montréal, Montréal, Québec, Canada

^2^School of Biological Sciences, Life Sciences Building, University of Bristol, Bristol, United Kingdom

^3^Department of Ecology, Faculty of Science, Charles University, Prague, Czech Republic

Author for correspondence: hedvig.nenzen@gmail.com

Supplementary Tables:

Table S1. Reference sources used for determining the presence of species in each of the 71 sites.

|  | Fossil site | Reference |
| --- | --- | --- |
| 1 | Huélago | Alberdi, MT, Alonso, MA, Azanza, B, Hoyos, M, Morales, J. 2001. Vertebrate taphonomy in circum-lake environments: three cases in the Guadix-Baza Basin (Granada, Spain). Palaeogeography, Palaeoclimatology, Palaeoecology, 165, 1-26. |
| 2 | Huéscar-1 | Alonso Diago, MA, Hoyos, M, Alberdi, MT. 2003. Tafonomía del yacimiento de vertebrados pleistoceno de Huéscar 1. Estudios geológicos, 59, 213-227. |
| 3 | Labeko Koba | Altuna, J, Mariezkurrena, K. 2000. Macromammals of the Labeko Koba site (Arrasate, Basque Country). Munibe, 52, 107-181. |
| 4 | Lezetxiki | Straus, LG.1976. Análisis arqueológico de la fauna paleolítica del norte de la Península Ibérica. Munibe, 4, 277-285. |
| 5 | La Parte | Alvarez-Lao, D, Arbizu, M, Carrocera, E. 2002. La Parte. Yacimiento Paleontológico Cuaternario. Siero. Consejerías de Educación y Cultura, y de Infraestructuras y Política Territorial. Gobierno del Principado de Asturias. |
| 6 | Cueva del Castillo | Pike-Tay, A, Cabrera Valdés, V, Bernaldo de Quirós, F. 1999. Seasonal variations of the Middle–Upper Paleolithic transition at El Castillo, Cueva Morín and El Pendo (Cantabria, Spain). Journal of Human Evolution, 36, 283–317 |
| 7 | Cueva Morín | Pike-Tay, A, Cabrera Valdés, V, Bernaldo de Quirós, F. 1999. Seasonal variations of the Middle–Upper Paleolithic transition at El Castillo, Cueva Morín and El Pendo (Cantabria, Spain). Journal of Human Evolution, 36, 283–317 |
| 8 | Cueto de la Mina | Stuart, AJ, Sulerzhitsky, LD, Orlova, LA, Kuzmin, YV, Lister, AM. 2002. The latest woolly mammoths (Mammuthus primigenius Blumenbach) in Europe and Asia: a review of the current evidence. Quaternary Science Reviews, 21, 1559-1569 |
| 9 | Los Torrejones | Arribas Herrera, A, Díez Fernández-Lomana, JC, Jordá Pardo, JF. 1995. Primeras ocupaciones en los depósitos Pleistocenos de la cueva de los Torrejones (Sistema Central Español, Tamajón, Guadalajara): litoestratigrafía y actividad biológica. Cuaternario y Geomorfología, 11, 55-66. |
| 10 | Fonelas 1 | Arribas, A, Riquelme, JA, Palmqvist, P, Garrido, G, Hernández, R, et. al. 2001. Un nuevo yacimiento de grandes mamíferos villafranquienses en la Cuenca de Guadix-Baza (Granada): Fonelas P-1, primer registro de una fauna próxima al límite Plio-Pleistoceno en la Península Ibérica. Boletín Geológico y Minero, 112 (4), 3-34. |
| 11 | Cueva del Congosto | Arribas Herrera, A, Jordá Pardo, JF. 1999. Los mamíferos del Cuaternario kárstico de Guadalajara (Castilla-La Mancha, España). In: La Huella del Pasado. Fósiles de Castilla-La Mancha (Aguirre, E. y Rábano, I. éds.), Patrimonio Histórico. Arqueología Castilla-La Mancha, 327-353. |
| 12 | Villacastín | Arribas Herrera, A. 1994. Paleontología de macromamíferos del yacimiento mesopleistoceno de villacastín (Segovia, España). Boletín Geológico y Minero. 105-4, 344-361. |
| 13 | Jarama VI | Arribas, A, Jordá Pardo, JF. 1999. Los mamíferos del Cuaternario kárstico de Guadalajara (Castilla-La Mancha, España). La Huella del Pasado. Fósiles de Castilla-La Mancha (ed. by E. Aguirre and I. Rábano), pp 327-353. Patrimonio Histórico. Arqueología Casti |
| 14 | Los Casares | Arribas, A, Jordá Pardo, JF. 1999. Los mamíferos del Cuaternario kárstico de Guadalajara (Castilla-La Mancha, España). La Huella del Pasado. Fósiles de Castilla-La Mancha (ed. by E. Aguirre and I. Rábano), pp 327-353. Patrimonio Histórico. Arqueología Casti |
| 15 | Buraca Escura | Aubry, T, Brugal, JP, Chauvière, FX I, G, Moura, MH, Plisson, H. 2001. modalités d'occupations au Plaeolithique supérieur dans la grotte de Buraca Escura (Redinha, Pombal, Portugal). Revista Portuguesa de Arqueologia, 4, 19-46. |
| 16 | Nerja | Riquelme Cantal, JA, Simón, M, Cortés, M. 2005. La fauna de mamíferos del Solutrense en la Cueva de Nerja. MUNIBE (Antropologia-Arkeologia), 57, Homenaje a Jesús Altuna, 255-263. |
| 17 | El Esquilleu | Baena Preysler J, Carrión, E, Ruiz, B, Ellwood B, Sesé C, et al. 2005. Paleoecología y comportamiento humano durante el Pleistoceno Superior en la comarca de Liébana: La secuencia de la Cueva de El Esquilleu (Occidente de Cantabria, España). In: Actas de la reunión Científica: Neandertales Cantábricos. Estado de la cuestión. Monografías del Museo Nacional y Centro de Investigación de Altamira, Santander. 20, 461-487. |
| 18 | Almonda | Cardoso, JL. 1997. As Grutas, os grandes mamíferos e o homem paleolótico: uma aproximaçao integrada ao território Portugês. Estudos do Quaternário, 1, 13-23. |
| 19 | Cabeça do Morto | Cardoso, JL. 1997. As Grutas, os grandes mamíferos e o homem paleolótico: uma aproximaçao integrada ao território Portugês. Estudos do Quaternário, 1, 13-23. |
| 20 | Casal do Torquato | Cardoso, JL. 1997. As Grutas, os grandes mamíferos e o homem paleolótico: uma aproximaçao integrada ao território Portugês. Estudos do Quaternário, 1, 13-23. |
| 21 | Cassals Robustos | Cardoso, JL. 1997. As Grutas, os grandes mamíferos e o homem paleolótico: uma aproximaçao integrada ao território Portugês. Estudos do Quaternário, 1, 13-23. |
| 22 | Condeixa | Cardoso, JL. 1997. As Grutas, os grandes mamíferos e o homem paleolótico: uma aproximaçao integrada ao território Portugês. Estudos do Quaternário, 1, 13-23. |
| 23 | Fontainhas | Cardoso, JL. 1997. As Grutas, os grandes mamíferos e o homem paleolótico: uma aproximaçao integrada ao território Portugês. Estudos do Quaternário, 1, 13-23. |
| 24 | Foz de Enxarrique | Cardoso, JL. 1997. As Grutas, os grandes mamíferos e o homem paleolótico: uma aproximaçao integrada ao território Portugês. Estudos do Quaternário, 1, 13-23. |
| 25 | Meirinha | Cardoso, JL. 1997. As Grutas, os grandes mamíferos e o homem paleolótico: uma aproximaçao integrada ao território Portugês. Estudos do Quaternário, 1, 13-23. |
| 26 | Molianos | Cardoso, JL. 1997. As Grutas, os grandes mamíferos e o homem paleolótico: uma aproximaçao integrada ao território Portugês. Estudos do Quaternário, 1, 13-23. |
| 27 | Porto Covo | Cardoso, JL. 1997. As Grutas, os grandes mamíferos e o homem paleolótico: uma aproximaçao integrada ao território Portugês. Estudos do Quaternário, 1, 13-23. |
| 28 | Santo Antao do Tojal | Cardoso, JL. 1997. As Grutas, os grandes mamíferos e o homem paleolótico: uma aproximaçao integrada ao território Portugês. Estudos do Quaternário, 1, 13-23. |
| 29 | Casa de Moura | Cardoso, JL. 1993. Contribuição para o conhecimento dos grandes mamíferos do Plistocénico Superior de Portugal. Câmara Municipal de Oeiras, Oeiras. 567 pp. |
| 30 | Columbeira | Cardoso, JL. 1993. Contribuição para o conhecimento dos grandes mamíferos do Plistocénico Superior de Portugal. Câmara Municipal de Oeiras, Oeiras. 567 pp. |
| 31 | Escoural | Cardoso, JL. 1993. Contribuição para o conhecimento dos grandes mamíferos do Plistocénico Superior de Portugal. Câmara Municipal de Oeiras, Oeiras. 567 pp. |
| 32 | Figueira brava | Cardoso, JL. 1993. Contribuição para o conhecimento dos grandes mamíferos do Plistocénico Superior de Portugal. Câmara Municipal de Oeiras, Oeiras. 567 pp. |
| 33 | Fontainhas | Cardoso, JL. 1993. Contribuição para o conhecimento dos grandes mamíferos do Plistocénico Superior de Portugal. Câmara Municipal de Oeiras, Oeiras. 567 pp. |
| 34 | Furninha | Cardoso, JL. 1993. Contribuição para o conhecimento dos grandes mamíferos do Plistocénico Superior de Portugal. Câmara Municipal de Oeiras, Oeiras. 567 pp. |
| 35 | Gruta das Salemas | Cardoso, JL. 1993. Contribuição para o conhecimento dos grandes mamíferos do Plistocénico Superior de Portugal. Câmara Municipal de Oeiras, Oeiras. 567 pp. |
| 36 | Lapa de Rainha | Cardoso, JL. 1993. Contribuição para o conhecimento dos grandes mamíferos do Plistocénico Superior de Portugal. Câmara Municipal de Oeiras, Oeiras. 567 pp. |
| 37 | Lorga de Dine | Cardoso, JL. 1993. Contribuição para o conhecimento dos grandes mamíferos do Plistocénico Superior de Portugal. Câmara Municipal de Oeiras, Oeiras. 567 pp. |
| 38 | Mealhada | Cardoso, JL. 1993. Contribuição para o conhecimento dos grandes mamíferos do Plistocénico Superior de Portugal. Câmara Municipal de Oeiras, Oeiras. 567 pp. |
| 39 | Pedreira das Salemas | Cardoso, JL. 1993. Contribuição para o conhecimento dos grandes mamíferos do Plistocénico Superior de Portugal. Câmara Municipal de Oeiras, Oeiras. 567 pp. |
| 40 | Pego do Diabo | Cardoso, JL. 1993. Contribuição para o conhecimento dos grandes mamíferos do Plistocénico Superior de Portugal. Câmara Municipal de Oeiras, Oeiras. 567 pp. |
| 41 | Santimamiñe | López-Quintana, JC, Guenaga-Lizasu, A. 2011. Revisión estratigráfica del depósito arqueológico de la cueva de Santimamiñe (Kortezubi, Bizkai): campañas de 2004 a 2006. Cronoestratigrafía y paleoambiente. In López Quintana,JC. (ed.) La cueva de Santimamiñe: revisión y actualización (2004-2006). Excavaciones arqueológicas en Bizkaia, Kobie 1: 7-70. |
| 42 | Grota do Caldeirao | Davis, SJM. 2002. The mammals and birds from the Gruta do Caldeirão, Portugal. Revista Portuguesa de Arqueologia, 5, 29-98. |
| 43 | Cueva de Bolomor | Fernandez Peris, J, Guillem, P, Fumanal, MP, Martinez, R. 1994. Cova del Bolomor (Tavernes de Valldigna, Valencia). Primeros datos de una secuencia del Pleistoceno medio. Saguntum, 27, Valencia, 9-37. |
| 44 | Lapa do Picareiro | Ferreira Bicho, N, Haws, JA, Hockett, B, Markova, A, Belcher, W. 2002. Paleoecologia e ocupação humana da Lapa do Picareiro: resultados preliminares. Revista Portuguesa de Arqueologia. 6 (49-81). |
| 45 | L'Arbreda | Estevez, J. 1987. La fauna de l'Arbreda (sector Alfa) en el conjunt de faunes del Plistocè Superior. Cypsela VI, 73-87. |
| 46 | Atapuerca-Dolina | Jan van der Made, personal communication |
| 47 | Atapuerca-Galería | Jan van der Made, personal communication |
| 48 | Algar de Cascais | Brugal, JP, Valente, MJ. 2004. Dynamic of large mammalian associations in the Pleistocene of Portugal. In: Actas do IV congresso de arquologia peninsular, Faro, Portugal. pp 15-27. |
| 49 | Algar de Joao Ramos | Brugal, JP, Valente, MJ. 2004. Dynamic of large mammalian associations in the Pleistocene of Portugal. In: Actas do IV congresso de arquologia peninsular, Faro, Portugal. pp 15-27. |
| 50 | Abauntz | Altuna, J, Mariezkurrena, K. 1982. Restos óseos del yacimiento prehistórico de Abauntz (Arraiz, Navarra). In: Trabajos de arqueología Navarra, 2, 347-353. |
| 51 | Axlor | Jesús González Urquijo, Juan José Ibáñez Estévez, Joseba Ríos Garaizar 2004. Excavación arqueológica en el yacimiento de Axlor (Dima, Bizkaia). MEMORIA. 20 pp. |
| 52 | Amalda | Yravedra, J. 2010. A Taphonomic Perspective on the Origins of the Faunal Remains from Amalda Cave (Spain). Journal of Taphonomy, 8, 301-334. |
| 53 | Cueva de Mollet | Maroto, J, Julià, R, López-García, JM, Blain, H-A. 2012. Chronological and environmental context of the Middle Pleistocene human tooth from Mollet Cave (Serinyà, NE Iberian Peninsula). Journal of Human Evolution 62, 655-663. |
| 54 | Cueva Negra | Walker, MJ, López-Martínez, M, Carrión-García, JS, Rodríguez-Estrella, T, San-Nicolás del-Toro, M, et al. 2012. Cueva Negra del Estrecho del Río Quípar (Murcia, Spain): A late Early Pleistocene hominin site with an “Acheulo-Levalloiso-Mousteroid” Palaeolithic assemblage. Quaternary International, 294, 135-159. |
| 55 | Cúllar de Baza | Alberdi, MT, Alonso, MA, Azanza, B, Hoyos, M, Morales, J. 2001. Vertebrate taphonomy in circum-lake environments: three cases in the Guadix-Baza Basin (Granada, Spain). Palaeogeography, Palaeoclimatology, Palaeoecology, 165, 1-26. |
| 56 | Sierra de Quibas | Montoya, P, Alberdi, MR, Blázquez, AM, Barbadillo, LJ, Fumanal, MP, van der Made, J, et. al. 1999. La Fauna del Pleistoceno Inferior de la Sierra de Quibas (Abanilla, Murcia). Estudios Geologicos, 55, 127-161. |
| 57 | Bora gran d'en carreres | García, N, Arsuaga, JL. 2003. Last Glaciation cold-adapted faunas in the Iberian Peninsula. Deinsea, 9, 159-170. |
| 58 | Prado Vargas | De Torres Pérez-Hidalgo, T, Rodríguez Nuere, B, González Alvaro, P. 1986. Excavación de la Cueva de Prado Vargas, Cornejo (Burgos-España), 281-289. |
| 59 | Bolinkoba | Castanos, P. 1983. Estudio de los macromamíferos del yacimiento prehistórico de Bolinkoba (Abadiano-Vizcaya). Kobie 13, 261-298, Diputación Foral de Bizkaia, Bilbao. |
| 60 | Aitzbitarte III | Stage Three Project http://www.esc.cam.ac.uk/research/research-groups/oistage3 |
| 61 | Cova Negra | Stage Three Project http://www.esc.cam.ac.uk/research/research-groups/oistage3 |
| 62 | Cueva de El Pendo | Stage Three Project http://www.esc.cam.ac.uk/research/research-groups/oistage3 |
| 63 | Devil's Tower | Stage Three Project http://www.esc.cam.ac.uk/research/research-groups/oistage3 |
| 64 | Ekain | Stage Three Project http://www.esc.cam.ac.uk/research/research-groups/oistage3 |
| 65 | Gorham's Cave | Stage Three Project http://www.esc.cam.ac.uk/research/research-groups/oistage3 |
| 66 | La Riera | Stage Three Project http://www.esc.cam.ac.uk/research/research-groups/oistage3 |
| 67 | Los Moros I | Stage Three Project http://www.esc.cam.ac.uk/research/research-groups/oistage3 |
| 68 | Reclau Viver | Stage Three Project http://www.esc.cam.ac.uk/research/research-groups/oistage3 |
| 69 | Roc de la Melca | Stage Three Project http://www.esc.cam.ac.uk/research/research-groups/oistage3 |
| 70 | Valina | Stage Three Project http://www.esc.cam.ac.uk/research/research-groups/oistage3 |
| 71 | Zafarraya | Stage Three Project http://www.esc.cam.ac.uk/research/research-groups/oistage3 |

Table S2. The species present (indicated by 1) at each period. Weight categories: 1 = < 45 kg, 2 = 45-90 kg, 3 = 90-360 kg, 4 = 360-1000 kg, 5 = >1000 kg. Phylogenetic replacement categories: 0 = Not replaced by a species in the same genus in the next time period, 1 = Replaced by a species in the next time period, 2 = Species still present.

| Species | Weight category | Early Pleistocene | Middle Pleistocene | Last Interglacial Maximum | Last Glacial Maximum | Holocene | Present | Replaced phylo-genetically |
| --- | --- | --- | --- | --- | --- | --- | --- | --- |
|  |  | 850 kyr BP | 450 kyr BP | 120 kyr  BP | 21 kyr  BP | 10 kyr BP | 0 kyr  BP |  |
| *Bison priscus* | 4 | 0 | 0 | 1 | 1 | 0 | 0 | 0 |
| *Bison schoetensacki* | 4 | 0 | 1 | 0 | 0 | 0 | 0 | 1 |
| *Bison voigtstedtensis* | 4 | 1 | 0 | 0 | 0 | 0 | 0 | 1 |
| *Bos primigenius* | 4 | 0 | 1 | 1 | 1 | 1 | 0 | 1 |
| *Bos taurus* | 4 | 0 | 0 | 0 | 0 | 0 | 1 | 2 |
| *Canis lupus* | 1 | 0 | 0 | 1 | 1 | 1 | 1 | 2 |
| *Canis mosbachensis* | 1 | 1 | 1 | 0 | 0 | 0 | 0 | 1 |
| *Capra alba* | 2 | 1 | 0 | 0 | 0 | 0 | 0 | 0 |
| *Capra pyrenaica* | 2 | 0 | 0 | 0 | 1 | 1 | 1 | 2 |
| *Capra sp.* | 1 | 0 | 0 | 1 | 0 | 0 | 0 | 1 |
| *Capreolus capreolus* | 1 | 0 | 0 | 1 | 1 | 1 | 1 | 2 |
| *Capreolus priscus* | 2 | 0 | 1 | 0 | 0 | 0 | 0 | 1 |
| *Cervus elaphus* | 3 | 1 | 1 | 1 | 1 | 1 | 1 | 2 |
| *Coelodonta antiquitatis* | 5 | 0 | 0 | 0 | 1 | 0 | 0 | 0 |
| *Crocuta crocuta* | 2 | 1 | 1 | 1 | 1 | 1 | 0 | 0 |
| *Cuon alpinus* | 1 | 0 | 1 | 0 | 0 | 0 | 0 | 0 |
| *Dama dama* | 2 | 0 | 1 | 1 | 1 | 0 | 1 | 2 |
| *Dama nestii* | 2 | 1 | 0 | 0 | 0 | 0 | 0 | 1 |
| *Elephas antiquus* | 5 | 1 | 1 | 1 | 0 | 0 | 0 | 0 |
| *Equus altidens* | 4 | 1 | 1 | 0 | 0 | 0 | 0 | 1 |
| *Equus asinus* | 3 | 0 | 0 | 0 | 0 | 0 | 1 | 2 |
| *Equus caballus* | 4 | 0 | 0 | 0 | 0 | 0 | 1 | 2 |
| *Equus ferus* | 4 | 0 | 0 | 1 | 1 | 1 | 0 | 1 |
| *Equus hydruntinus* | 3 | 0 | 0 | 1 | 1 | 0 | 0 | 1 |
| *Eucladoceros giulii* | 3 | 1 | 0 | 0 | 0 | 0 | 0 | 0 |
| *Gulo gulo* | 1 | 0 | 0 | 0 | 1 | 0 | 0 | 0 |
| *Hemitragus bonali* | 3 | 0 | 1 | 0 | 0 | 0 | 0 | 0 |
| *Hippopotamus amphibius* | 5 | 0 | 0 | 1 | 0 | 0 | 0 | 0 |
| *Hippopotamus antiquus* | 5 | 1 | 0 | 0 | 0 | 0 | 0 | 1 |
| *Hippopotamus sp.* | 5 | 0 | 1 | 0 | 0 | 0 | 0 | 1 |
| *Homo antecessor* | 2 | 1 | 0 | 0 | 0 | 0 | 0 | 1 |
| *Homo heidelbergensis* | 2 | 0 | 1 | 0 | 0 | 0 | 0 | 1 |
| *Homo neanderthalensis* | 2 | 0 | 0 | 1 | 0 | 0 | 0 | 1 |
| *Homo sapiens* | 2 | 0 | 0 | 0 | 1 | 1 | 1 | 2 |
| *Homotherium latidens* | 3 | 1 | 1 | 0 | 0 | 0 | 0 | 0 |
| *Hyaena hyaena* | 1 | 0 | 1 | 0 | 0 | 0 | 0 | 0 |
| *Mammuthus meridionalis* | 5 | 1 | 0 | 0 | 0 | 0 | 0 | 1 |
| *Mammuthus primigenius* | 5 | 0 | 0 | 1 | 1 | 0 | 0 | 0 |
| *Mammuthus trogontherii* | 5 | 0 | 1 | 0 | 0 | 0 | 0 | 1 |
| *Megaloceros giganteus* | 4 | 0 | 0 | 1 | 1 | 0 | 0 | 0 |
| *Megaloceros solilhacus* | 4 | 1 | 1 | 0 | 0 | 0 | 0 | 1 |
| *Ovibos moschatus* | 3 | 0 | 0 | 0 | 1 | 0 | 0 | 0 |
| *Ovis aries* | 1 | 0 | 0 | 0 | 0 | 0 | 1 | 2 |
| *Ovis musimon* | 1 | 0 | 0 | 0 | 0 | 0 | 1 | 2 |
| *Panthera gombaszoegensis* | 2 | 1 | 1 | 0 | 0 | 0 | 0 | 1 |
| *Panthera spelaea* | 3 | 0 | 1 | 1 | 1 | 0 | 0 | 0 |
| *Panthera pardus* | 2 | 0 | 0 | 1 | 1 | 0 | 0 | 0 |
| *Praeovibos sp.* | 3 | 1 | 0 | 0 | 0 | 0 | 0 | 0 |
| *Rangifer tarandus* | 3 | 0 | 0 | 0 | 1 | 0 | 0 | 0 |
| *Rupicapra pyrenaica* | 1 | 0 | 0 | 0 | 1 | 1 | 1 | 2 |
| *Rupicapra sp.* | 1 | 0 | 0 | 1 | 0 | 0 | 0 | 1 |
| *Saiga tatarica* | 1 | 0 | 0 | 0 | 1 | 0 | 0 | 0 |
| *Soergelia minor* | 3 | 1 | 0 | 0 | 0 | 0 | 0 | 0 |
| *Stephanorhinus etruscus* | 5 | 1 | 0 | 0 | 0 | 0 | 0 | 1 |
| *Stephanorhinus hemitoechus* | 5 | 0 | 1 | 1 | 1 | 0 | 0 | 0 |
| *Sus scrofa* | 2 | 1 | 1 | 1 | 1 | 1 | 1 | 0 |
| *Ursus arctos* | 4 | 0 | 0 | 1 | 1 | 1 | 1 | 2 |
| *Ursus deningeri* | 4 | 0 | 1 | 0 | 0 | 0 | 0 | 1 |
| *Ursus dolinensis* | 4 | 1 | 0 | 0 | 0 | 0 | 0 | 1 |
| *Ursus spelaeus* | 4 | 0 | 0 | 1 | 1 | 0 | 0 | 1 |

Table S3. Prey weight classes. This information, together with spatio-temporal co-occurrence and actualism (see main text) was used to establish the links between species in the food web.

|  |  |  | Possible Prey Weight Categories | | | | | |  |
| --- | --- | --- | --- | --- | --- | --- | --- | --- | --- |
| Maximum Weight | Weight Category | Species | 45 | 90 | 180 | 360 | 1000 | 10000 | Reference |
| 45 | 1 | *Canis lupus* | 1 | 1 | 1 | 1 | 0,5 | 0 | [1,2] |
| 45 | 1 | *Canis mosbachensis* | 1 | 1 | 1 | 1 | 0,5 | 0 | [3] |
| 45 | 1 | *Cuon alpinus* | 1 | 1 | 1 | 0 | 0 | 0 | [4–6] |
| 45 | 1 | *Gulo gulo* | 0,5 | 0,5 | 1 | 1 | 0,5 | 0,5 | [7,8] |
| 45 | 1 | *Hyaena hyaena* | 1 | 0,5 | 0,5 | 0,5 | 0 | 0 | [8] |
| 90 | 2 | *Crocuta crocuta* | 1 | 1 | 1 | 0,5 | 0,5 | 0,5 | [10,11] |
| 90 | 2 | *Homo antecessor* | 1 | 1 | 1 | 1 | 1 | 1 | [12] |
| 90 | 2 | *Homo heildelbergensis* | 1 | 1 | 1 | 1 | 1 | 1 | [13] |
| 90 | 2 | *Homo neanderthalensis* | 1 | 1 | 1 | 1 | 1 | 1 | [13] |
| 90 | 2 | *Homo sapiens* | 1 | 1 | 1 | 1 | 1 | 1 |  |
| 80 | 2 | *Panthera gombaszoegensis* | 1 | 1 | 1 | 0,5 | 0,5 | 0,5 | [14] |
| 90 | 2 | *Panthera pardus* | 1 | 0,5 | 0,5 | 0 | 0 | 0 | [15] |
| 180 | 3 | *Homotherium latidens* | 0,5 | 0,5 | 1 | 1 | 1 | 0,5 | [16] |
| 360 | 4 | *Panthera spelaea* | 0,5 | 0,5 | 1 | 1 | 1 | 0,5 | [15,17] |
| 1000 | 5 | *Ursus arctos* | 0,5 | 0,5 | 0,5 | 0,5 | 0 | 0 | [18–20] |
| 1000 | 5 | *Ursus deningeri* | 0,5 | 0,5 | 0,5 | 0,5 | 0 | 0 | [21] |
| 1000 | 5 | *Ursus dolinensis* | 0,5 | 0,5 | 0,5 | 0,5 | 0 | 0 | [22] |

Supplementary Figures:


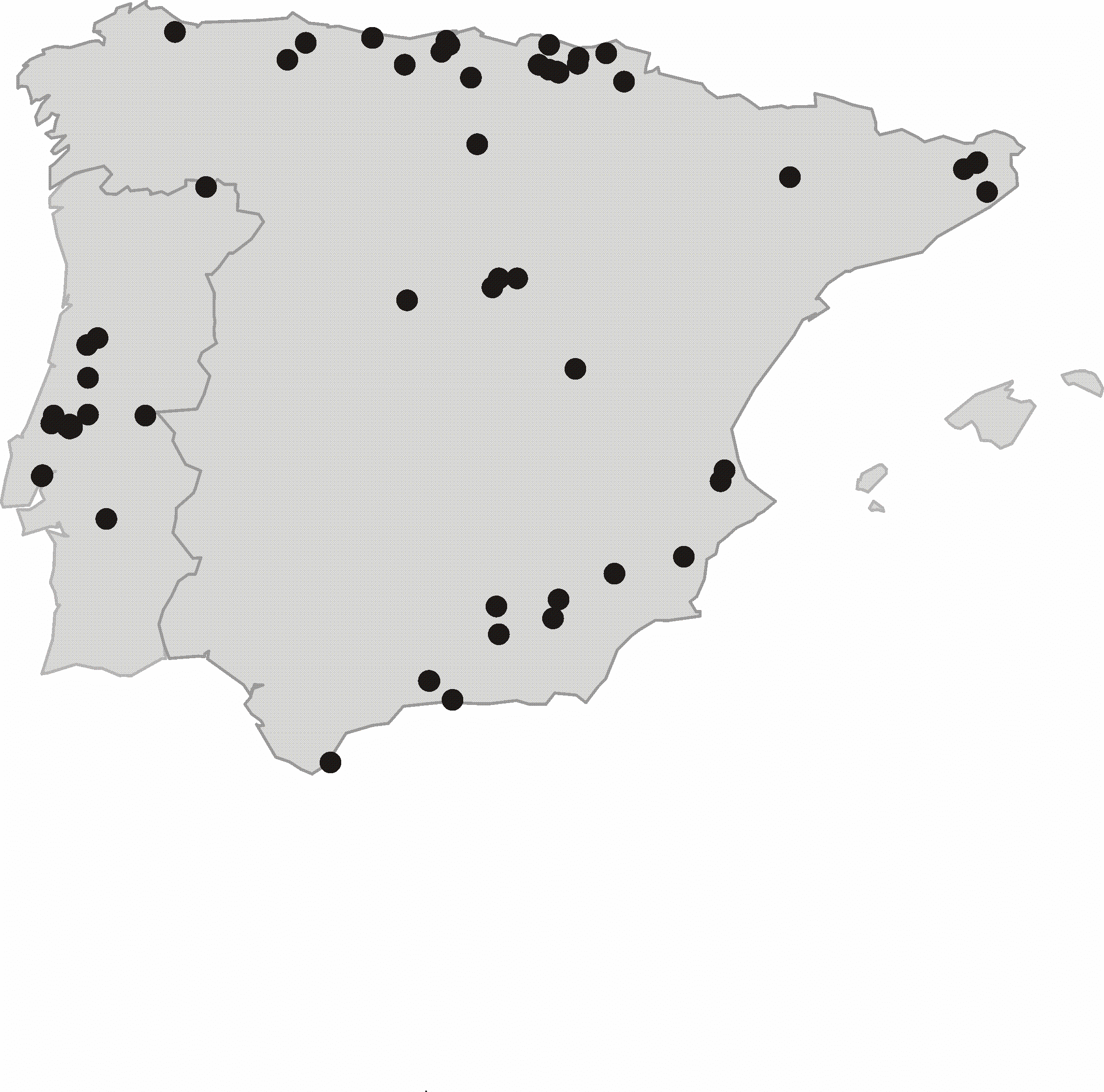


Figure S1. Geographic location on the Iberian Peninsula of the Quaternary fossil sites used for constructing the ancient food webs.


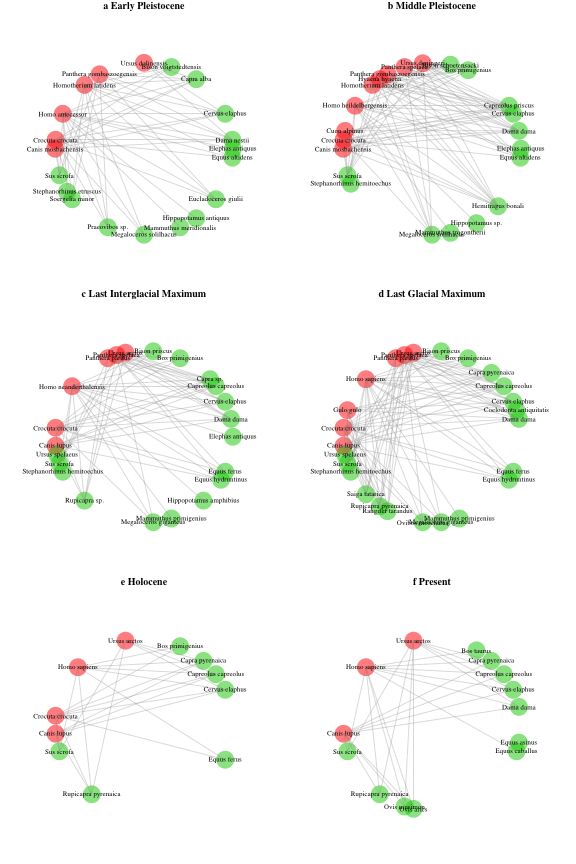


Figure S2. Large mammal food webs of the Iberian Peninsula during the Quaternary. (a) Early Pleistocene (850,000 years before present, or 850 ky BP); (b) Middle Pleistocene (450 ky BP); (c) Last Interglacial Maximum (120 ky BP); (d) Last Glacial Maximum (21 ky BP); (e) Early Holocene (10 ky BP); and (f) Present. Each node (green for prey, red for predator) is a species, and each link indicates a trophic interaction.


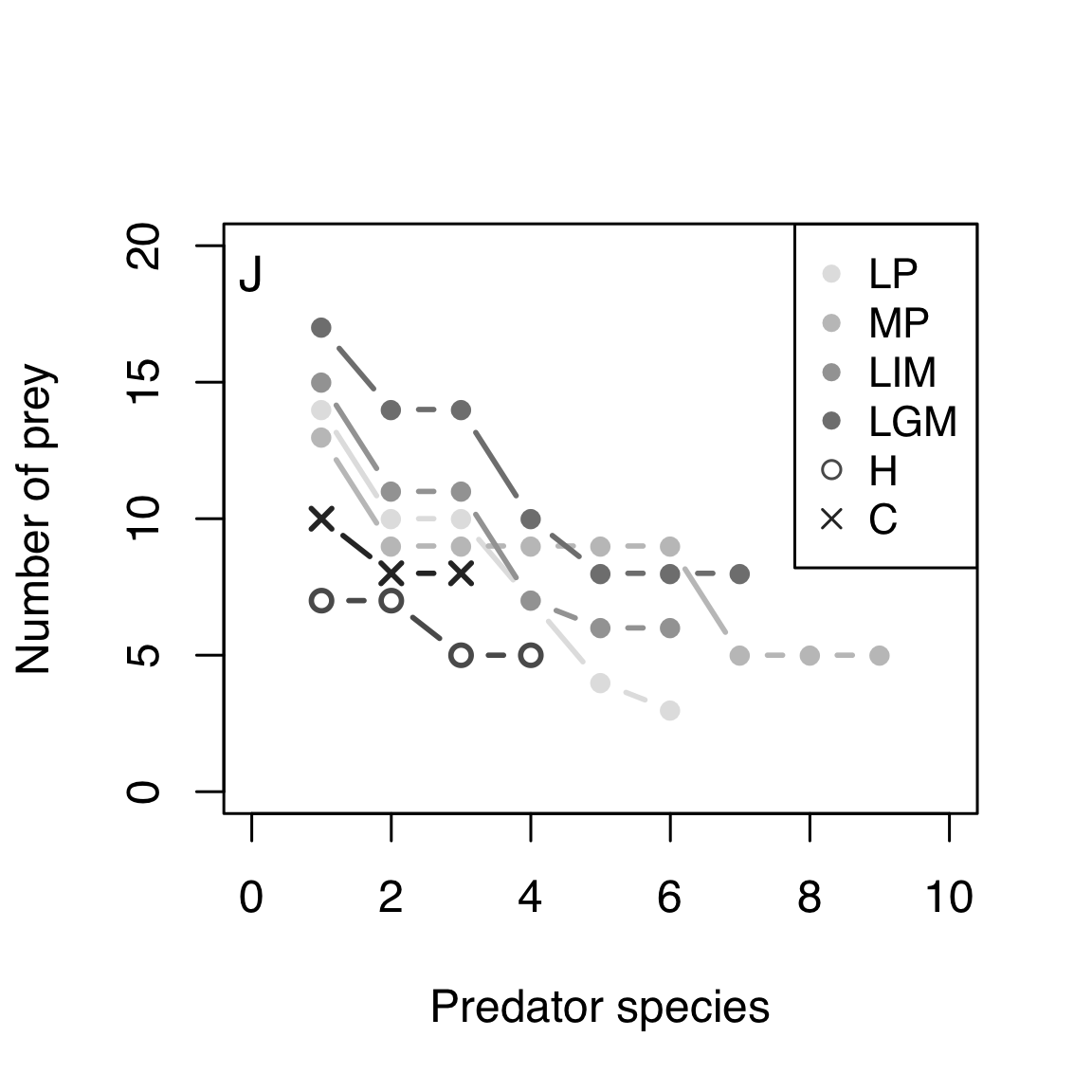


Figure S3. Number of prey per predator in each time period, with species sorted in descending order. The time periods are the same as in Figure 1.


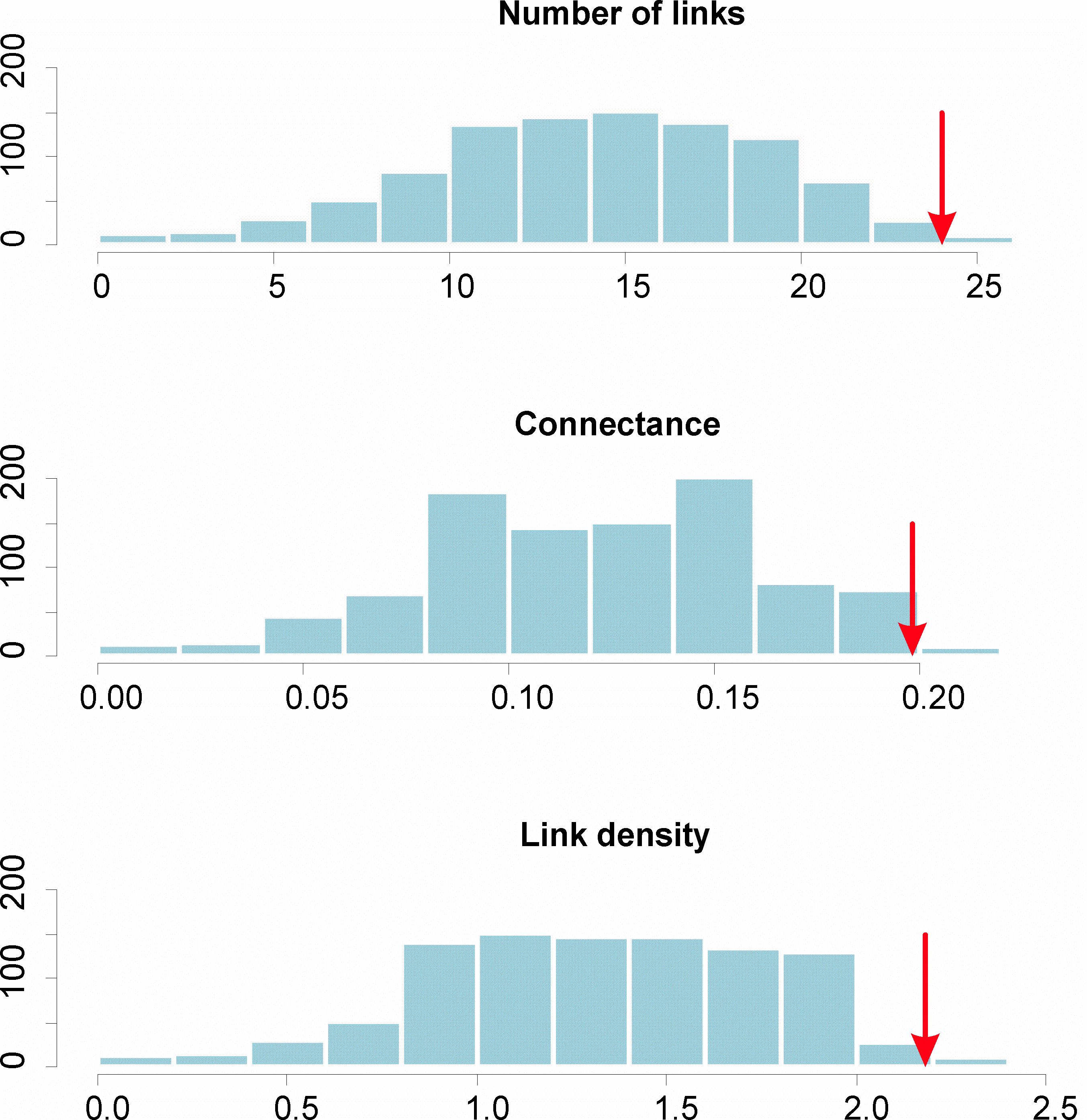


Figure S4. A random extinction experiment demonstrate that the distribution of the number of links, connectance and link density observed Holocene food web is not expected by chance. 1000 food webs have been created extracting randomly 11 species from last glacial maximum food web (the observed number of extinct species in the Holocene).

**Supplementary References**

1 Sidorovich, V. E., L.L., T. & Jedrzejewska, B. 2003 Wolf Canis lupus numbers, diet and damage to livestock in relation to hunting and ungulate abundance in northeastern Belarus during 1990-2000. *Wildlife Biol.* **9**, 103–111.

2 Stahler, D. R., Smith, D. W. & Guernsey, D. S. 2006 Foraging and Feeding Ecology of the Gray Wolf (*Canis lupus*): Lessons from Yellowstone National Park, Wyoming, USA. *J. Nutr.* **136**, 1923S–1926S.

3 Tedford, R. H., Wang, X. & Taylor, B. E. 2009 Phylogenetic Systematics of the North American Fossil Caninae (Carnivora: Canidae). *Bull. Am. Museum Nat. Hist.* **243**, 1–218. (doi:10.1206/574.1)

4 Grassman, L. I., Tewes, M. E., Silvy, N. J. & Kreetiyutanont, K. 2005 Spatial ecology and diet of the dhole *Cuon alpinus* (Canidae, Carnivora) in north central Thailand. *Mammalia* **69**. (doi:10.1515/mamm.2005.002)

5 Thinley, P., Kamler, J. F., Wang, S. W., Lham, K., Stenkewitz, U. & Macdonald, D. W. 2011 Seasonal diet of dholes (*Cuon alpinus*) in northwestern Bhutan. *Mamm. Biol. - Zeitschrift für Saugetierkd.* **76**, 518–520. (doi:10.1016/j.mambio.2011.02.003)

6 Kamler, J., Johnson, A., Vongkhamheng, C. & Bousa, A. 2012 The diet, prey selection, and activity of dholes (*Cuon alpinus*) in northern Laos. *J. Mammal.* **93**, 627–633. (doi:10.1644/11-mamm-a-241.1)

7 Gustavsen, L. 2006 The diet of a facultative scavenger, the wolverine (*Gulo gulo*), after recolonization of wolf (*Canis lupus*) in southern Norway.

8 Yeakel, J. D., Guimarães, P. R., Bocherens, H. & Koch, P. L. 2013 The impact of climate change on the structure of Pleistocene food webs across the mammoth steppe. *Proc. B.* **280**, 2013239.

9 Leakey, L., Milledge, S., Leakey, S., Edung, J., Haynes, P., Kiptoo, D. K. & McGeorge, A. 1999 Diet of striped hyaena in northern Kenya. *Afr. J. Ecol.* **37**, 314–326.

10 Mills, M. G. L. 1984 Prey selection and feeding habits of the large carnivores in the southern Kalahari. *Koedoe* **27**, 281–294.

11 Hayward, M. W. 2006 Prey preferences of the spotted hyaena (*Crocuta crocuta*) and degree of dietary overlap with the lion (*Panthera leo*). *J. Zool.* **270**, 606 – 614. (doi:10.1111/j.1469-7998.2006.00183.x)

12 Van der Made, J. 2001 The ungulates from Atapuerca: Stratigraphy and biogeography. *Anthropologie* **105**, 95–113.

13 Bocherens, H. 2010 Stable isotopes in collagen and Late Quaternary carnivore palaeoecology. Geophysical Research Abstracts, 12. *Geophys. Res. Abstr.* **12**.

14 Palmqvist, P., Pérez-Claros, J. A., Janis, C. M. & Gröcke, D. R. 2008 Tracing the ecophysiology of ungulates and predator–prey relationships in an early Pleistocene large mammal community. *Palaeogeogr. Palaeoclimatol. Palaeoecol.* **266**, 95–111. (doi:10.1016/j.palaeo.2008.03.015)

15 Owen-Smith, N. & Mills, M. G. L. 2008 Predator-prey size relationships in an African large-mammal food web. *J. Anim. Ecol.* **77**, 173–83. (doi:10.1111/j.1365-2656.2007.01314.x)

16 Palmqvist, P., Martínez-Navarro, B. & Arribas, A. 1996 Prey Selection by Terrestrial Carnivores in a Lower Pleistocene Paleocommunity. *Paleobiology* **22**, 514–534.

17 Hayward, M. W. & Kerley, G. I. H. 2005 Prey preferences of the lion (*Panthera leo*). *J. Zool.* **267**, 309–322. (doi:10.1017/S0952836905007508)

18 Mattson, D. J. 1997 Use of ungulates by Yellowstone grizzly bears *Ursus arctos*. *Biol. Conserv.* **81**, 161–177.

19 Persson, I. L., Wikan, S., Swenson, J. E. & Mysterud, I. 2001 The diet of the brown bear *Ursus arctos* in the Pasvik Valley, northeastern Norway. Wildlife Biology, 7, 27-37. *Wildlife Biol.* **7**, 27–37.

20 Richards, M. P., Pacher, M., Stiller, M., Quilès, J., Hofreiter, M., Constantin, S., Zilhão, J. & Trinkaus, E. 2008 Isotopic evidence for omnivory among European cave bears: Late Pleistocene *Ursus spelaeus* from the Peştera cu Oase, Romania. *Proc. Natl. Acad. Sci.* **105**, 600–604. (doi:10.1073/pnas.0711063105)

21 García García, N., Feranec, R. S., Arsuaga, J. L., Bermúdez de Castro, J. M. & Carbonell, E. 2009 Isotopic analysis of the ecology of herbivores and carnivores from the Middle Pleistocene deposits of the Sierra De Atapuerca, northern Spain. *J. Archaeol. Sci.* **36**, 1142–1151. (doi:10.1016/j.jas.2008.12.018)

22 Olive, F. 2006 Evolution of Plio Pleistocene larger Carnivores in Africa and Western Europe. Anthropologie, 110, 850-869. *Anthropologie* **110**, 850–869.
